# Clearance from the mouse brain by convection of interstitial fluid towards the ventricular system

**DOI:** 10.1186/s12987-015-0019-5

**Published:** 2015-10-05

**Authors:** Beatrice Bedussi, Monique G. J. T. B. van Lier, Jonas W. Bartstra, Judith de Vos, Maria Siebes, Ed VanBavel, Erik N. T. P. Bakker

**Affiliations:** Department of Biomedical Engineering and Physics, Academic Medical Center, Room L0-119. Meibergdreef 9, PO Box: 22660, 1105 AZ Amsterdam, The Netherlands

**Keywords:** Interstitial fluid, Cerebrospinal fluid, Ventricular system, Paravascular space, Glymphatic pathway

## Abstract

**Background:**

In the absence of a true lymphatic system in the brain parenchyma, alternative clearance pathways for excess fluid and waste products have been proposed. Suggested mechanisms for clearance implicate a role for brain interstitial and cerebrospinal fluids. However, the proposed direction of flow, the anatomical structures involved, and the driving forces are controversial.

**Methods:**

To trace the distribution of interstitial and cerebrospinal fluid in the brain, and to identify the anatomical structures involved, we infused a mix of fluorescent tracers with different sizes into the cisterna magna or striatum of mouse brains. We subsequently performed confocal fluorescence imaging of horizontal brain sections and made 3D reconstructions of the mouse brain and vasculature.

**Results:**

We observed a distribution pattern of tracers from the parenchyma to the ventricular system, from where tracers mixed with the cerebrospinal fluid, reached the subarachnoid space, and left the brain via the cribriform plate and the nose. Tracers also entered paravascular spaces around arteries both after injection in the cisterna magna and striatum, but this appeared to be of minor importance.

**Conclusion:**

These data suggest a bulk flow of interstitial fluid from the striatum towards the adjacent lateral ventricle. Tracers may enter arterial paravascular spaces from two sides, both through bulk flow from the parenchyma and through mixing of CSF in the subarachnoid space. Disturbances in this transport pathway could influence the drainage of amyloid β and other waste products, which may be relevant for the pathophysiology of Alzheimer’s disease.

**Electronic supplementary material:**

The online version of this article (doi:10.1186/s12987-015-0019-5) contains supplementary material, which is available to authorized users.

## Background

Brain function is critically dependent on the extracellular fluids that surround the neurons. Interstitial fluid (ISF) is in direct contact with neuronal cells and delivers nutrients and oxygen, while at the same time it removes waste products. Brain ISF is derived from water and solutes that enter through the blood–brain barrier. This process allows specific ion transporters and channels to regulate its composition [1]. Next to ISF and blood plasma, cerebrospinal fluid (CSF) forms the third extracellular fluid compartment of the brain. The composition of CSF differs minimally from ISF, which suggests a relatively free communication between CSF and ISF. Although recent work identified lymphatic vessels in the dura mater [2, 3], the brain parenchyma lacks a true lymphatic system. CSF may take over this role in fluid balance and waste removal, by drainage of excess fluid from arachnoid granulations, the aforementioned dural lymphatics, and other pathways. The classical view is that CSF is mainly secreted by specialized vascular structures, the choroid plexuses [4]. The CSF subsequently flows through the ventricular system of the brain, which consists of the two lateral ventricles, the third ventricle, and the fourth ventricle, which finally connects to the subarachnoid space. However, recent insights suggest that CSF and ISF physiology is much more complex than previously believed [5, 6]. Thus, CSF and ISF may exchange over the ependymal epithelium that covers the ventricular system [7, 8], and CSF may also reenter the parenchyma along paravascular pathways [4, 6, 9]. In addition, ISF may drain along basement membranes of capillaries and arteries, retrograde to the direction of blood flow [10]. Understanding the drainage pathways of the brain is required for unraveling the removal mechanisms of toxic waste products such as amyloid β, as well as for developing targeted delivery of treatment modalities. Therefore, the current study set out to investigate clearance from the brain. In particular, we focused on paravascular pathways as a clearance mechanism. To this extent we infused tracers in the ISF (striatum) and CSF compartment (cisterna magna, CM) and studied their distribution by confocal imaging of brain sections and 3D reconstruction of the whole mouse head using an imaging cryomicrotome.

## Methods

### Animals

For this study, 20 animals were included, 10 for injection in the striatum (7 for confocal microscopy, 3 for 3D cryomicrotome imaging) and 10 for injection of tracers in the cisterna magna (5 for confocal microscopy, 5 for 3D cryomicrotome imaging). Male C57BL/6JOlaHsd mice were obtained from Harlan (The Netherlands). Animals were fed ad libitum with standard laboratory food and free access to water. They were allowed to acclimatize for at least 1 week before being enrolled in experimental protocols. All experimental protocols were approved by the Committee for Animal Experiments of the Academic Medical Center Amsterdam, according to the EU guidelines.

### Anesthesia

In all experiments mice were anesthetized with a mixture of ketamine (126 mg/kg, Nimatek, Eurovet, Bladel, The Netherlands) medetomidine (0.2 mg/kg, Dormitor, Orion Pharma, Mechelen, Belgium) and atropine (0.5 mg/kg, Atropinesulfaat, Eurovet) in PBS (Phosphate Buffered Saline, Lonza, Basel, Switzerland) by intraperitoneal injection (75 μl/10 g body weight). To prevent swelling of the brain, 0,2 mg/ml dexamethason (Sigma-Aldrich, Zwijndrecht Nederland) in 20 % ethanol (10 μl/10 g body weight) was added to the anesthetic mixture. Experiments were carried during daytime hours, i.e. in the sleeping phase of the mouse diurnal rhythm.

### Reagents

Dextran, Texas Red-labeled (3 kD, Ex. 595 nm/Em. 615 nm) and dextran, Fluorescein-labeled (500 kD, Ex. 494 nm/Em. 521), both lysine-fixable, were purchased from Molecular Probes-Life Technologies (Eugene, OR, USA). For immunofluorescence, brains were embedded in Tissue-Tek (Sakura, Leiden, The Netherlands) before freezing and sectioning. Cell nuclei were stained with VECTASHIELD Mounting Medium with DAPI (4, 6-diamidino-2-phenylindole, Burlingame, CA, USA) according to the manufacturer’s protocol. Anti-Laminin antibody (Sigma-Aldrich) was used to visualize the vasculature.

### Experimental procedure

When the animals were completely anesthetized, their heads were shaved. Next, the animals were fixed in a stereotactic frame (lab standard stereotaxic, Stoelting, Dublin, Ireland), while the head was kept at the same level as the body. To maintain physiological body temperature, animals were placed on a heating pad. Additional oxygen (99 %) was provided to prevent hypoxia and ocular lubricant ophthalmic (DURATEARS, Alcon, Breda, The Netherlands) was used to keep the eyes hydrated. In this study we used two different injection sites: the cisterna magna and the striatum. For cisterna magna injections, after separating the subcutaneous tissues and the midline of the neck muscle, a 29-gauge needle was placed into the cistern and connected to a polyethylene catheter. A total volume of 5 μl of a mixture (1:1) of both dextrans, 3 kD Texas Red (10 g/l), and 500 kD dextran, Fluorescein (10 g/l), was infused at a controlled flow rate of 0.17 μl/min using a syringe pump (Harvard Apparatus, Holliston, MA, USA) over a 30 min period. For injection into the striatum (coordinates from the Bregma 0.5 mm rostral, 1.5 mm lateral, and 2.5 mm deep), animals were completely anesthetized, the skull was exposed and after additional local anesthesia (Xylocaine 10 %, AstraZeneca BV, Zoetermeer, The Netherlands), a small burr hole was drilled using a dental drill. Subsequently, a custom-made 33-gauge polyethylene catheter was inserted into the brain using the stereotactic device. For this site of injection we anticipated that the maximum volume tolerated is smaller than in the cisterna magna. For this reason, 2 μl of the dextran mixture was infused at a controlled flow rate of 0.066 μl/min, over a 30 min period. In both protocols, animals were euthanized with an overdose of anesthetic 30 min after start of the infusion. Needles were removed after death.

### Confocal microscopy

After sacrifice of the animal the brains were dissected, embedded in Tissue-Tek and frozen in liquid nitrogen prior to storage at −80 °C. Samples were cut longitudinally with a microtome (5 μm slices). We used 3.7 % paraformaldehyde to fix the slices and mounted them with Vectashield mounting medium containing DAPI nuclear stain. Images were acquired using a Confocal Laser Scanning Microscope (Leica TCS SP8, Buffalo Grove, IL, USA). A 10× oil immersion was used for the overviews and a 20× oil immersion objective was used to image the details.

## 3D cryomicrotome imaging

After surgery, animals were flushed with PBS to remove the blood in the vasculature prior to vascular filling with the replica material via the abdominal aorta. To fill the vasculature we used fluorescent (UV blue, excitation 375 nm, emission 505 nm, VasQtec, Zurich, Switzerland) replica material (Batson’s #17, Polysciences, Eppelheim, Germany). After filling and polymerization, the specimens were frozen at −20 °C for at least 24 h and then the heads were cut with a guillotine. The frozen specimens were embedded in a black gel (carboxymethylcellulose sodium solvent 5 %—Brunschwig Chemie, Amsterdam, The Netherlands– mixed with Indian ink 5 %—Royal Talens, Apeldoorn, The Netherlands), and placed at −20 °C. Each sample was cut from nose to the second cervical vertebrae using 50 μm slice thickness for the nose until the start of the brain and 10 μm for the remaining part of the head, using an imaging cryomicrotome (custom made, Amsterdam, The Netherlands) [11, 12]. After each slice a white light picture of the sample and episcopic fluorescent images for the Dextrans, 5 kD Texas Red (exitation 577 nm, emission 635 nm), and 500 kD Dextran, Fluorescein (exitation 480 nm, emission 535 nm) and vascular cast were recorded (excitation 365 nm, emission 505 nm) using a 4096 × 4096, 16 bit resolution digital camera (Apogee Alta U-16, Oceanside, CA, USA) equipped with a variable- focus lens (Nikon 70–180 mm, Amsterdam, The Netherlands). The resulting registered stack of sequential images contained on average 2200 images per color with an in-plane resolution of 5 μm.

### Image processing

For visualizing purposes, two adjacent slices were combined using maximal intensity projection and converted to 8 bit greyscale images. The head was reconstructed in 3D using Amira (Visage Imaging GmbH, Berlin, Germany). This yielded a detailed 3D virtual representation of the mouse head vasculature with co-localized dextran deposition.

## Results

### Tracers move from the striatum into the ventricles via ISF

**Fig. 1:**
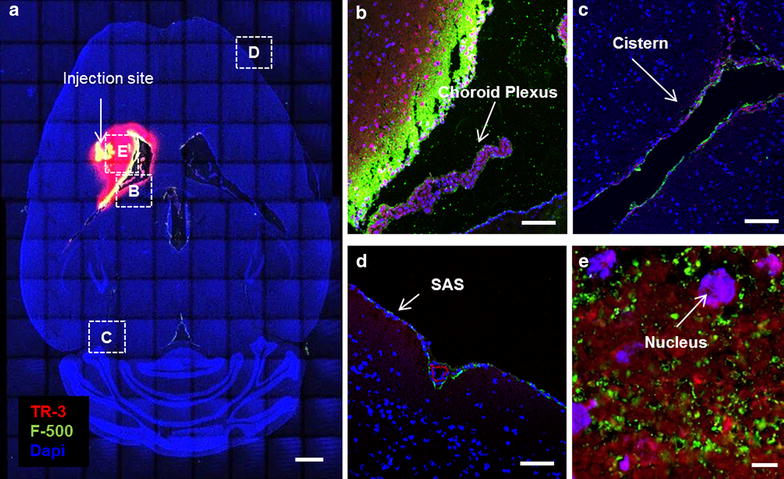
Distribution of the tracers (TR-3 and F-500) 30 min after injection in the striatum. **a** Tile scan of a *horizontal* brain section at −2 mm from the bregma. A distinct distribution of the tracers towards the lateral ventricle was observed. *Scale bar* 1 mm. Anatomical details are shown in *panels* *B*–*E*. **b** Ventricle and choroid plexus. Tracers coming from the parenchyma reached the ventricle, crossing the ependymal layer. TR-3 is taken up by the choroid plexus while F-500 stays on its *outer layer*. *Scale bar* 100 µm. **c** The ambient cistern showed both tracers. *Scale bar* 100 µm. **d** The subarachnoid space showed both F-500 and TR-3, although the signal of TR-3 is weaker compared to F-500. Note the autofluorescence of elastin (*red*) in the meningeal artery. *Scale bar* 100 µm. *Panel* **e** Detail of the brain parenchyma. TR-3 is observed intra- and intercellular, while F-500 appears to remain extracellular. *Scale bar* 10 µm

To investigate clearance pathways in the brain interstitium, we infused a mixture of a low molecular weight tracer, TR-3, and a high molecular weight tracer, F-500, in the striatum of mice (n = 7). We used this combination of high and low molecular weight tracers to estimate the respective contributions of diffusion and bulk flow to dispersion, as there should be a ~fourfold difference in diffusion distance of these tracers based on their diffusion coefficients in the brain [13]. We analyzed the distribution of the tracers by confocal fluorescence imaging of horizontal brain sections. This showed that the tracers had spread from the infusion site through the parenchyma towards the closest lateral ventricle (Fig. 1). The co-localization of the dyes, the directionality of this transport and the distance traveled indicate that this transport is dominated by convection. F-500 followed intercellular spaces from the site of injection to the wall of the ventricle. There, it crossed the ependymal layer and was detected in the ventricular cavity. TR-3 followed the same route, but was also taken up by parenchymal cells along the way towards the ventricle. Both tracers were detected further along the ventricular system, reaching the 3rd and 4th ventricle. In some experiments the tracers had reached the downstream subarachnoid space. Detailed images show the presence of both tracers in the lateral ventricle (B), cisterns (C), and the subarachnoid space (SAS; D). Figure 1e shows that TR-3 entered parenchymal cells, while F-500 appeared to remain confined to the extracellular spaces.

### The low molecular weight tracer penetrates the choroid plexus

**Fig. 2:**
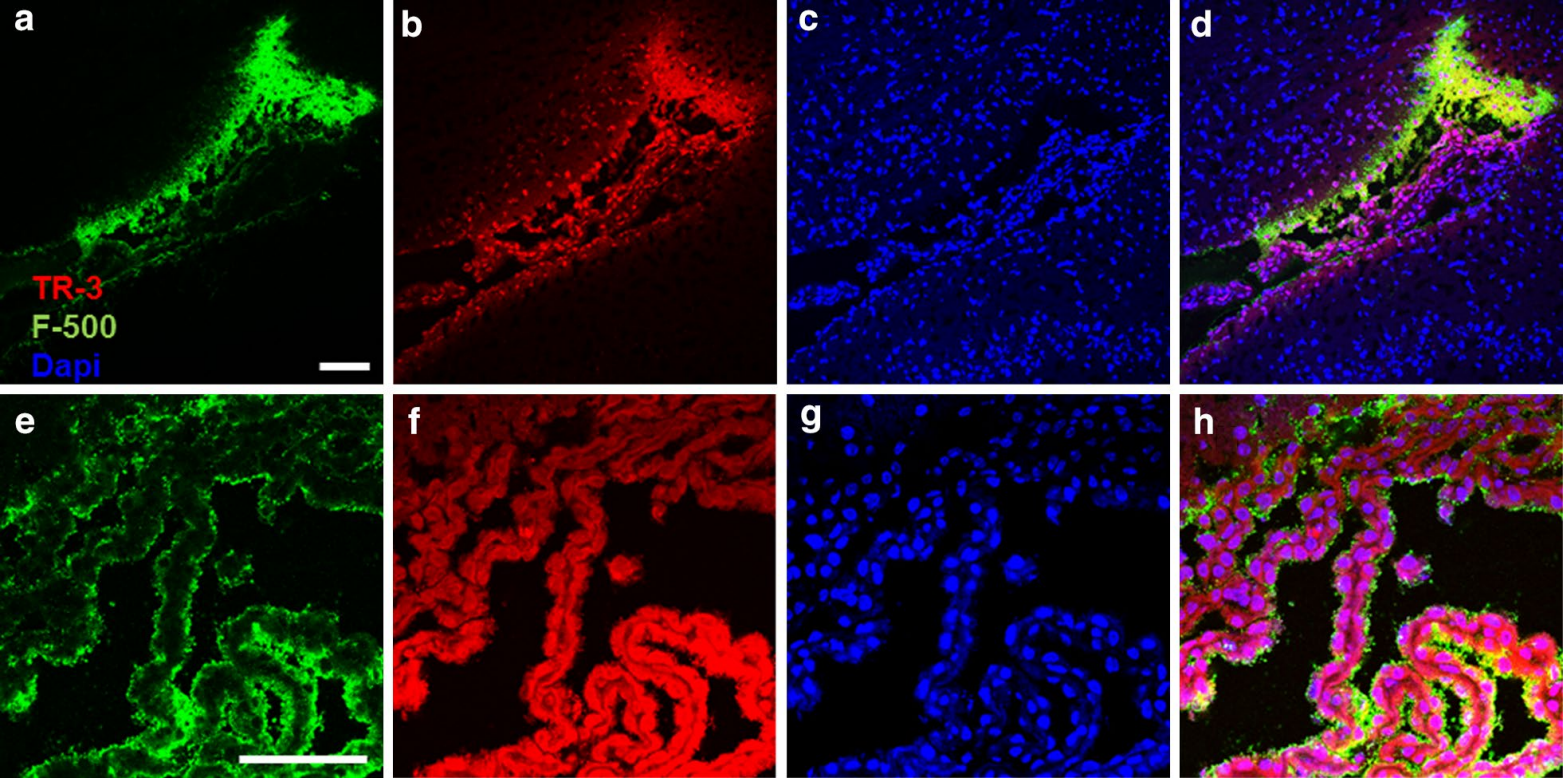
Penetration of TR-3 in the choroid plexus after striatum injection. *Horizontal section* −3 mm from the bregma. **a**–**d** Overview of the lateral ventricle. F-500 accumulated towards the ependymal layer (**a**) while TR-3 appeared to cross the ependymal more easily (**b**). Cell nuclei stained with DAPI (**c**). A merged picture is shown in *panel* **d**. **e**–**g** Detail of the choroid plexus. F-500 remains confined to the surface of the choroid plexus (**e**) while TR-3 clearly penetrated the choroid plexus (**f**). Cell nuclei stained with DAPI (**g**). Merged picture (**h**). *Scale bar* 100 µm

After infusion in the striatum, we observed that both tracers crossed the ependymal cells into the ventricle. However, particularly F-500 accumulated in the parenchyma close to the ependyma, suggesting it acts as a partial barrier for this tracer (Fig. 2a–d). Such accumulation, in the course from the injection site to the ventricle, is a strong additional indication for bulk flow from the parenchyma of the striatum. We also observed a bright signal for TR-3 in the choroid plexuses. TR-3 clearly penetrated the epithelial cells of the choroid plexus while F-500 was confined to extracellular side of the same cells (Fig. 2e–h).

## 3D reconstruction of blood vessels and tracer transport in the mouse’s head after infusion in the striatum

**Fig. 3:**
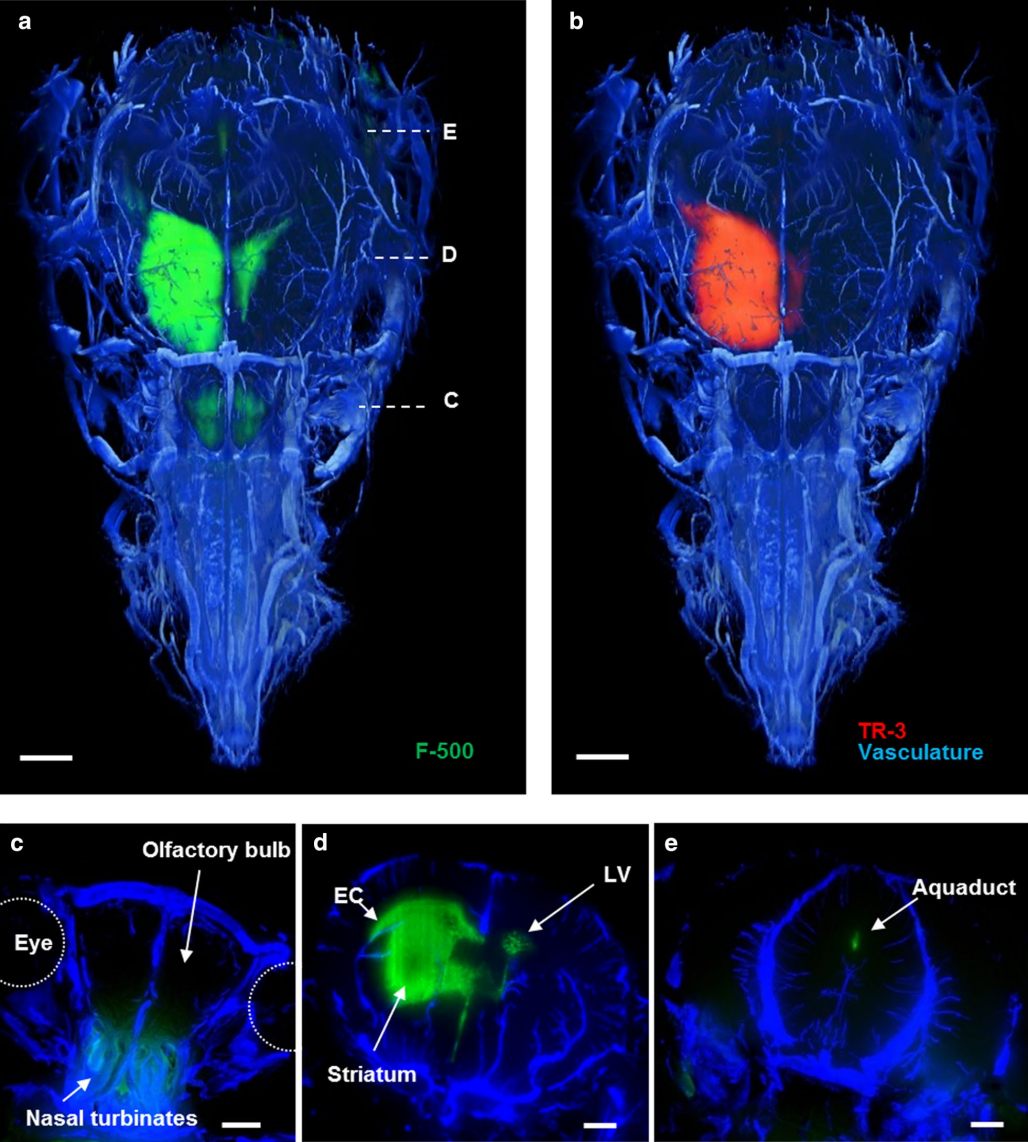
Three dimensional reconstruction of the vasculature and distribution of tracers in a mouse head after injection in the striatum. **a** F-500 is present at the injection site, the lateral ventricles and in the olfactory bulb. **b** TR-3 is present in the same compartments but could not be detected in the olfactory bulb. *Panels* *C*–*E* represent maximum intensity projections of 50 images at the indicated levels shown in **a** of the vasculature and F-500. **c** F-500 reached the nasal turbinates through the cribriform plate. **d** F-500 distributed in the striatum, reached the external capsule (*EC*), and the contralateral ventricle (*LV*) via the ventricular system. **e** The aqueduct of sylvius showed F-500. *Panels* **a**, **b**: dorsal view. Panels **c**–**e**: coronal view. *Scale bar* 1 mm

3D images of tracer distribution after injection in the striatum were generated by an imaging cryomicrotome. After sacrifice of the animal, blood vessels were filled with fluorescent cast material via the aorta, in order to map tracer distribution against the vasculature. The whole mouse head was sectioned (approximately 2500 sections) after freezing. En face fluorescent images were taken during automated sectioning. This approach preserves dye present in fluid compartments (ISF and CSF), which could be lost during sectioning and processing. In addition, since we included the whole mouse head, it also captured the drainage pathways from the brain to peripheral structures. Figure 3 shows tracer distribution after 30 min continuous infusion in the striatum. Similar to the confocal images, in the 3D reconstruction we observed that tracers drained from the striatum to the adjacent lateral ventricle. 2D coronal views of the brain (Fig. 3c–e) show the presence of F-500 further along the external capsule, in the ventral side of the olfactory bulb, the nasal turbinates, as well as the contralateral ventricle and third ventricle (Additional file 1). Similar to F-500, TR-3 was observed in the parenchyma close to the injection site, from where it followed the same route towards the ventricular system (not shown). Thus, these images confirmed the observations made with confocal imaging on tracer clearance from the parenchyma by bulk flow of ISF towards the ventricles.

### Tracers injected into the cisterna magna disperse over the subarachnoid space

**Fig. 4:**
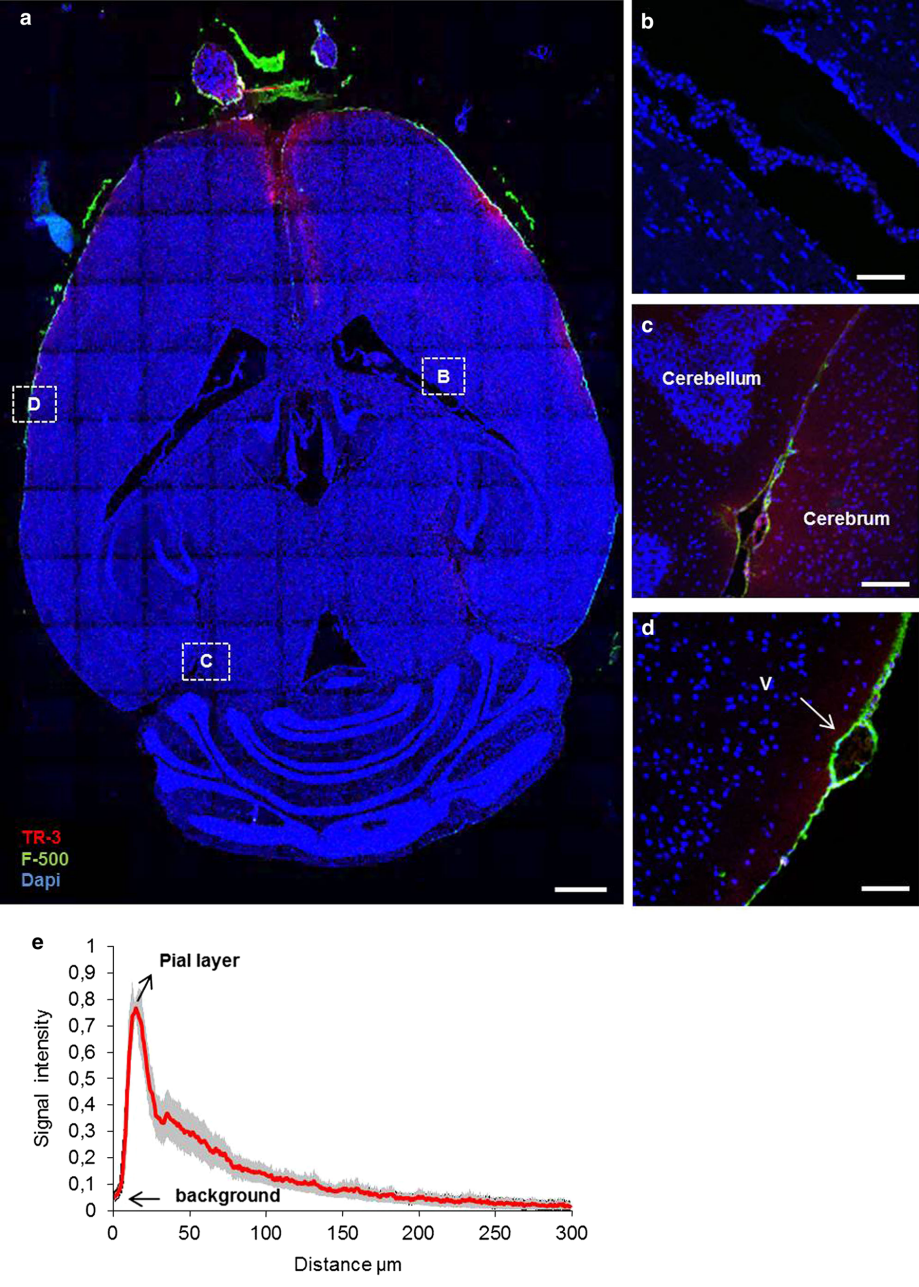
Distribution of the tracers 30 min after injection in the cisterna magna. **a** Tile scan of a *horizontal section* −2 mm from the bregma. The ventricular system showed no signal, while the olfactory bulb and the subarachnoid space showed both tracers. TR-3 and F-500 were observed in the subarachnoid space along the entire section. *Scale bar* 1 mm. Anatomical details are shown in *panels* *B*–*D*. **b** The ventricle and choroid plexus did not show TR-3 and F-500. **c** The ambient cistern showed both tracers, with TR-3 also entering the adjacent parenchyma. **d** A leptomeningeal vein (v, *arrow*) in the SAS showed F-500 in the paravascular space. *Scale bar* 100 µm. **e** Signal intensity as a function of distance for TR-3 from the SAS to the brain parenchyma across the pial layer, 30 min after CM injection. The mean distance is 64 ± 7 µm (N = 5)

We next evaluated bulk flow using tracer infusion in the cisterna magna, using the same mix of tracers as injected into the striatum (n = 5). Again we used a 30 min continuous infusion, to map the distribution of tracers from their injection site. Confocal imaging showed that after 30 min of infusion, both tracers distributed over the subarachnoid space and entered the large cisterns of the brain. As shown in the overview (Fig. 4a) and in detail in panel b, the ventricles showed no signal, indicating that the tracers did not enter the ventricular system, neither along the CSF pathways nor via transport through the cortex, interstitial compartments and ventricular ependyma. Other views (Fig. 4c, d) showed that F-500 remained confined to the subarachnoid space, whereas TR-3 also penetrated the parenchyma, crossing the pia mater. This is particularly evident close to the tentorium cerebelli, and around the olfactory bulb. Analysis of TR-3 penetration across the pial layer indicated that this tracer was detected up to ~200 µm into the cortex (Fig. 4e). The average penetration depth was 64 µm.

### Reconstruction of the mouse head in 3D after tracer infusion in the cisterna magna

**Fig. 5:**
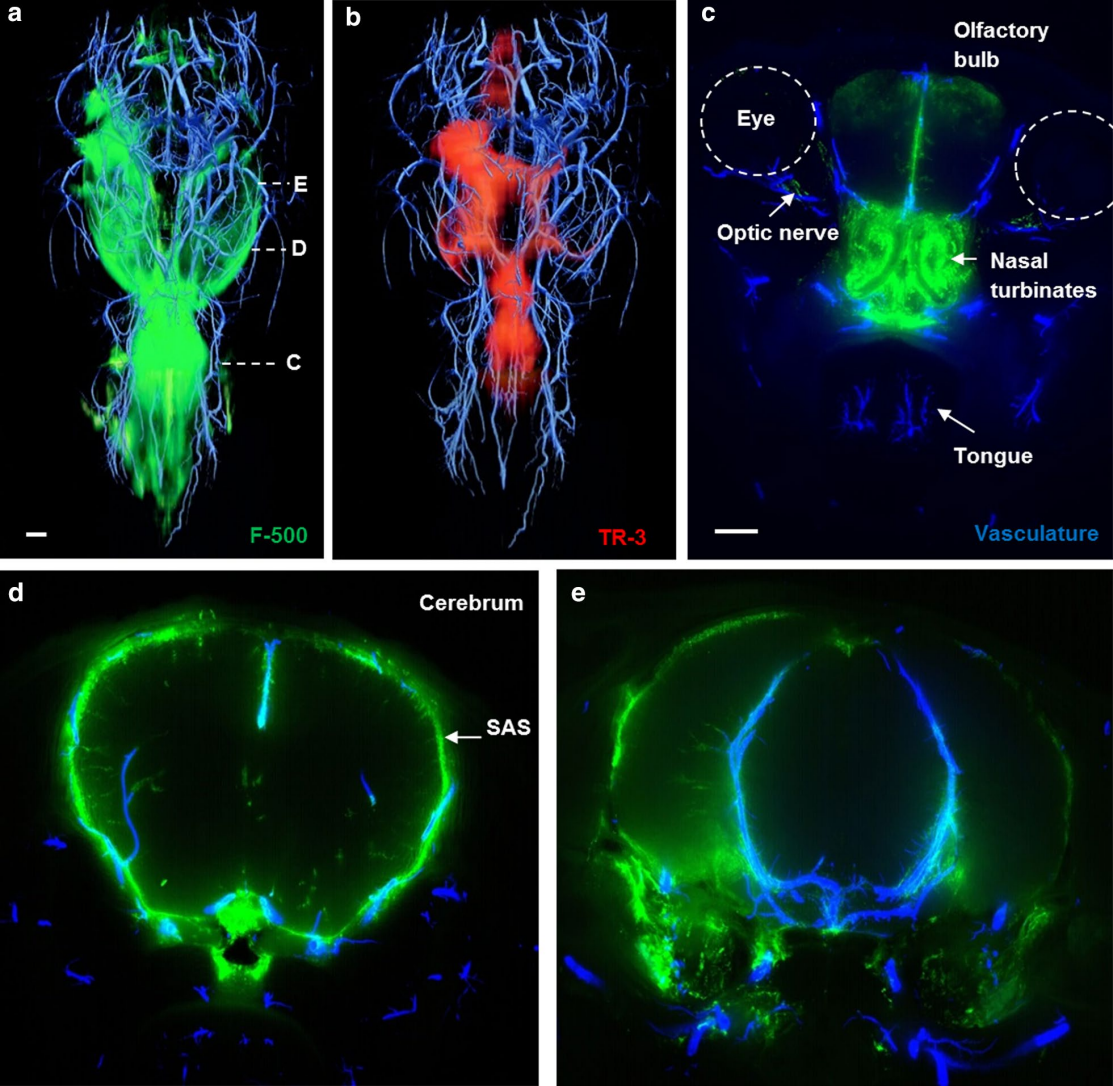
Three dimensional reconstruction of the vasculature with co-localized distribution of tracers in a mouse head after injection in the cisterna magna. **a** F-500 was found in the subarachnoid space and along the penetrating arteries of the brain. **b** TR-3 was observed in the same compartment, but less confined and also diffusely present in the brain parenchyma. This was particularly evident around the large arteries of the circle of Willis. **c** Outside the brain, a strong signal was found in the nasal turbinates and along the optic nerves. Maximum intensity projections of 50 images at the indicated levels of the vasculature and F-500 are shown in *panels* *C*–*E*. *Panels* **a**, **b**: dorsal view; **c**–**e**: coronal view. *Scale bar* 1 mm

We next studied 3D tracer distribution after injection of tracers in the cisterna magna. Figure 5a, b show the vasculature (in blue) and the tracers (F-500 in green and TR-3 in red); Fig. 5c–e show coronal views. The 3D image confirmed the observations made with confocal imaging (Additional file 2). Thus, most of the tracers were confined to the subarachnoid space, with a stronger signal for both tracers on the ventral side of the brain (Fig. 5e). Additionally, the dye was found in the longitudinal fissure, and around the major vessels arising from the cortex and ventral side of the brain (Fig. 5c–e). Interestingly, outside the brain the strongest signal for both was observed in the nose (Fig. 5c). The continuous presence of both dyes between the olfactory bulb and the nose suggest that these tracers leave the brain through the cribriform plate (Fig. 5c). The ventricular system was completely devoid of tracers (Fig. 5d). TR-3 showed the same distribution pattern, but more diffuse and less intense (not shown).

### Role of blood vessels in clearance

**Fig. 6:**
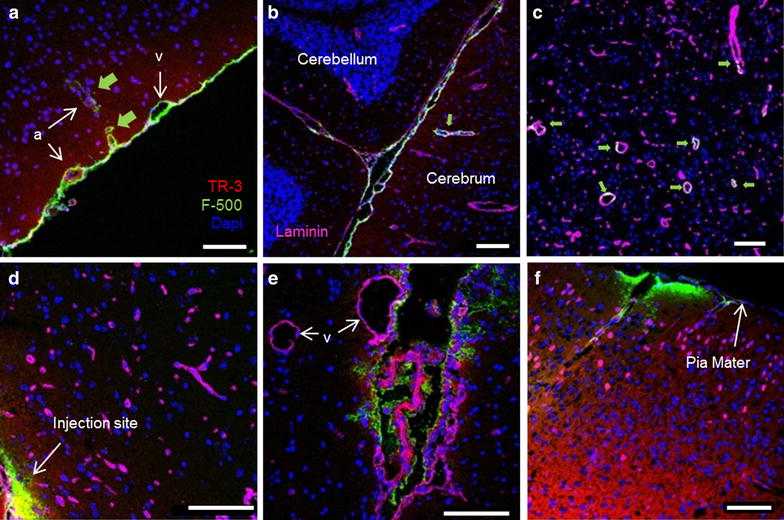
Role of the vasculature in bulk flow. **a**–**c** Cisterna magna injection, **d**–**f** striatum injection. Additional laminin staining is present in *panels* **b**–**f**. **a** Tracers were found in the subarachnoid space, embedding arteries (*a*) and veins (*v*). Arteries showed additional *red* autofluorescence from elastin. *Green arrows* F-500 followed paravascular spaces along arteries into the parenchyma. **b** F-500 was prominent in cisterns, where large arteries entered the brain (*green arrow*). **c** F-500 entered the paravascular space of vessels from the ventral side of the brain (*green arrows*). **d** Small vessels close to the injection site did not show tracers. **e** Choroid plexus with TR-3 and F-500, adjacent veins (*v*) did not show tracers. **f** Both tracers followed blood vessels towards the brain surface. F-500 accumulated at the pia mater. *Scale bar* 100 µm

**Fig. 7:**
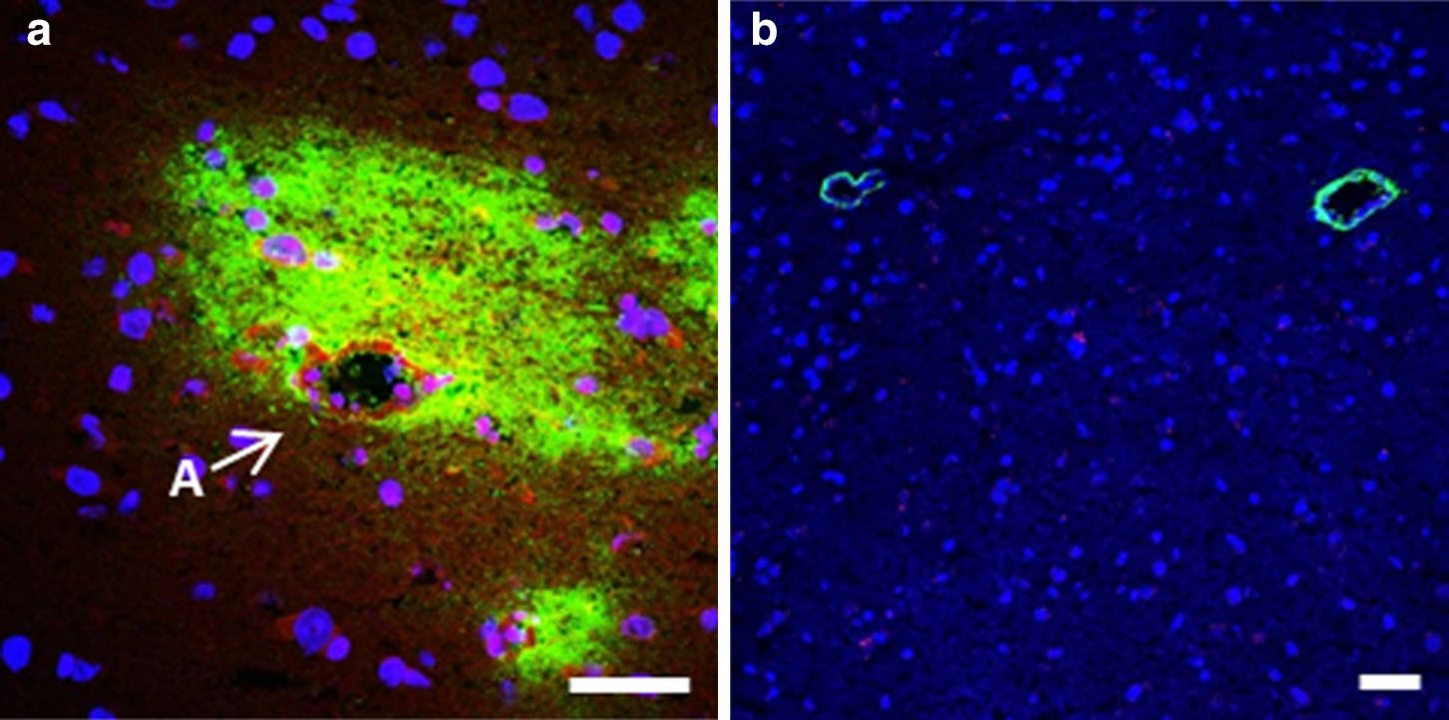
Different appearance of tracers around parenchymal vessels according to the injection site. **a** A case where tracers accumulated around an artery (*A*), after injection in the striatum. TR3 was taken up by smooth muscle ce6lls and parenchymal cells, whereas F500 was not. **b** Two parenchymal vessels showing F500 after injection in the cisterna magna. In this case the tracer entered the paravascular space from the CSF, but did not enter the parenchyma. We speculate that in *panel* **a** tracers were carried by bulk flow towards the artery, whereas in *panel* **b** the tracer entered the paravascular space as a result of the continuity with the SAS and the strong mixing action caused by pulsations in the CSF. *Scale bar* 50 µm

To further analyze the involvement of the vasculature and its peri- and/or paravascular spaces as potential distribution pathways, we stained sections with an anti-laminin antibody. Laminin-positive staining was observed both within the walls of blood vessels (arteries, veins, capillaries) and the meninges. Among blood vessels, large arteries could easily be discriminated from veins, particularly in the SAS, based on the autofluorescence of elastin (Fig. 6a). Following injection into the cisterna magna (CM), tracers were seen around both arteries and veins in the SAS, with penetration of dye along the vessel walls of arteries into the parenchyma (Fig. 6a). There was no visible boundary or difference in signal intensity between the paravascular signal and the subarachnoid space, suggesting that the subarachnoidal and paravascular spaces form a continuous fluid compartment (Fig. 6a). Tracers were most clearly visible in the ambient cistern where also several large arteries enter the brain (Fig. 6b). Note that TR-3 penetrated the parenchyma of the cerebrum but not the cerebellum. Within the parenchyma of the cerebrum, F-500 was observed along vessels that penetrate the brain from the ventral side (Figs. 6c, 7). Based on wall thickness, these vessels were most likely arteries. Following injection into the striatum, neither capillaries, nor veins close to the injection site in the striatum (Fig. 6d), nor the parenchymal vessels close to the lateral ventricle (Fig. 6e) showed tracers. However, occasionally tracers were seen to accumulate towards arteries ventral to the injection site and along the vessel walls near the cortex (Fig. 6f). In this case, TR-3 was seen along the vessel walls, while F-500 accumulated towards the pia mater. These observations suggest bulk flow of ISF close to the surface of the cortex towards the SAS.

## Discussion

In this study we investigated clearance from the brain and the role of peri- and paravascular pathways herein. The perivascular route is considered to be located along basement membranes within the vessel wall [10]. On the other hand, the paravascular space is located in between the brain parenchyma (astrocyte endfeet) and the vessel, and is filled with CSF [9]. The contribution of diffusion and bulk flow in the removal of metabolites and waste from brain extracellular fluids is a longstanding debate that recently has regained interest. Based on earlier work from Cserr [14–16], Rennels [4], Weller [10], and others, Abbott [6] concluded that ISF is not a static fluid, although a later review by Sykova and Nicholso [13] concludes that bulk flow is likely to be restricted to perivascular spaces and the main mechanism for exchange in the brain extracellular space is diffusion. More recent work of the Nedergaard group [9, 17, 18] suggests that paravascular pathways provide an influx route for CSF along arteries, which mixes with ISF and leaves the brain via venous outflow pathways. However, as pointed out by Hladky and Barrand [19], this view is at variance with many earlier studies and awaits further confirmation.

### Bulk flow from the striatum towards the ventricular system

The current study presents several arguments for the presence of bulk flow of interstitial fluid from the striatum towards the ventricles. The first evidence is the transport of the tracers after injection into the striatum, where the tracers distribute from the injection towards the nearest lateral ventricle. Both F-500 and TR-3 moved along extracellular spaces, albeit that TR-3 was also taken up by parenchymal cells, in accordance with in vitro data [20]. Such transport could theoretically be caused by diffusion, convection, or both. Discrimination between these processes is possible based on the distances traveled and the directionality of such transport. F-500 would be expected to diffuse a far smaller distance than TR-3 which, in our hands, was not the case. In addition, F-500 clearly moved towards the ventricles, and accumulated strongly at the ependymal layer. The accumulation is suggestive of a sieving process, where ISF is transported to the ventricle and part of the tracer stays behind. For the small TR-3, transport distances for diffusion would still be in the order of magnitude that we found [13], but also here the tracer preferentially moved towards the ventricles. This dye accumulated to a lesser extent, reflecting the possibility that it passes the ependymal layer more easily than F-500. Taken together, this suggests a significant contribution of tracer drainage by ISF bulk flow from the striatum into the ventricular system. In the lateral ventricle, ISF mixes with CSF once it passes the ependymal cells at the ventricle edge. Then, tracers follow CSF bulk flow along the ventricular system and get further distributed over the subarachnoid space.

### The choroid plexus as a potential exit route from the CSF

Floating in the ventricles, the choroid plexus is considered as the main source of CSF. In this study, we found that TR-3 penetrates into the choroid plexus. Distances are short, so penetration could be the result of diffusion from the ventricular CSF. Alternatively, it has been shown that active transport can be bi-directional over the epithelial cells of the choroid plexus [21]. The epithelial cells of the choroid plexus constitute the blood-CSF barrier (BCSFB) that limits the movement of solutes from blood to the CSF and vice versa. However, like the blood–brain barrier, the BCSFB contains transport systems for influx and/or efflux of nutrients, metabolic products and ions, and might also be involved in detoxification processes [21, 22]. This also opens the possibility that the choroid plexus may function as an exit route for waste products from the CSF. Indeed, ex vivo data show the removal of amyloid β from the choroid plexus [23] and in vivo, there is a correlation between choroid plexus dysfunction and amyloid β removal [24]. Nevertheless, the current data did not allow us to discriminate between diffusion and active transport of this particular tracer over the BCSFB.

### Lack of evidence for bulk flow from the subarachnoid space and paravascular spaces into the brain parenchyma

CSF and ISF may not only communicate at the ependymal layer of the ventricles, but also at the pial level [16]. In our experiments, after injection into the cisterna magna, the mixture of fluorescent tracers spread in the subarachnoid space. We observed that TR-3 entered the parenchyma close to the SAS and arteries, while F-500 was confined to the SAS and paravascular spaces. The TR-3 signal decreased steeply in deeper cortex layers, and was detected up to about 0.2 mm deep. Based on the diffusion coefficient for TR-3 in brain tissue [25], and an average diffusion time of 15 min in our experiments, one would estimate that this tracer may diffuse over an average distance of ~0.31 mm. This is more than the average distance that we observed: ~0.064 mm. Therefore, the penetration of tracer appears to be less than can be anticipated on the basis of diffusion. In fact, the small penetration depth suggests that diffusion is possibly hindered by bulk flow in the opposite direction. Thus, we suggest that in the current settings, there was limited penetration of small solutes from the SAS into the brain by diffusion (Fig. 4), and no bulk flow from the SAS and paravascular spaces into the brain parenchyma. This is further substantiated by the distribution of the high molecular tracer along the paravascular spaces. Thus, F-500 showed a steep decrease in intensity along arteries entering the brain, while a constant or even an increased concentration of dye would be expected due to a sieving action with paravascular inflow of CSF. A more likely explanation for the distribution of tracers over the SAS and along paravascular spaces could therefore be the strong mixing movement in the subarachnoid space due to arterial pulsations [26].

### Bulk flow of ISF: a continental divide

The current study suggests the presence of bulk flow of ISF from the striatum toward the ventricular system. We found no evidence for a contribution of recirculation or re-entrance from the sub-arachnoid or para-vascular spaces to this ISF flow. At this point, our interpretation differs from the glymphatic concept [9]. In particular, we did not observe outflow along parenchymal veins towards the subarachnoid space. Leptomeningeal veins may have shown tracers simply because the tracers are present in the SAS. Rather, the picture that emerges from our data is that ISF flow is generated by the transport of fluid from the capillaries to the interstitium. In other organs, such fluid transport is believed to be a very local phenomenon. According to the Starling balance of hydrostatic and oncotic pressures, fluid leaves higher pressure capillaries, close to the arterioles, and is mostly reabsorbed by venous capillaries. The remainder is removed by the lymphatic system. In the brain, fluid transport from capillaries to interstitium depends on active transport of ions and solutes rather than Starling forces, and reabsorption is thought to be absent [19]. Such fluid therefore needs to find its way to the CSF compartment. This would generate a cumulating flow, small in regions far from the ventricles (like rivulets high up in the mountains), and much larger when approaching the ventricles. Occasionally (Fig. 6f) we observed that tracer present close to the cortical surface finds its way to the brain surface along blood vessels. These findings resemble the observations by Weller [10]. We therefore speculate that there is a ‘continental divide’, a point of highest interstitial pressure where generated ISF flows to either the ventricles or the cortex. We suggest that in the mouse this watershed is situated very close to the cortical surface. Indeed, Arbel-Ornath [27] found that when tracers are injected in the cortex, they quickly drain out of the brain along arteries. In this analogy, peri- and paravascular spaces could be seen as fjords, allowing fluid transport from the mountains to the sea. It should be mentioned that the concept of fluid generation from capillaries is controversial [28–31], and we see the above view as a working hypothesis. Many issues remain to be addressed, including the nature and regulation of water transport over the BBB, the amount of generated ISF/CSF volume as compared to production by the choroid plexus, the interstitial pressure profile, relevance for removal of toxic products, and relevance for other species and conditions, notably for humans.

### Limitations

Injection of bulk fluid may be a confounding factor when studying interstitial fluid transport. The contribution of the infusion rate to tracer distribution is difficult to establish. We used an infusion rate far less than many theoretical [32] and experimental studies [10], but the contribution of the pumping rate still may not be completely ignored. In pilot studies we varied infusion rates both for the striatum and cisterna magna infusions, and found no clear differences in tracer distribution. Even when the infusion rate was reduced to zero, allowing only diffusion from the tip of the needle, fluorescein (0.38 kD) was found to subsequently drain from the striatum towards the lateral ventricle. Alternatively, increased perfusion rates and/or volumes applied to cisterna magna infusion did not appreciably alter the distribution of the tracers (data not shown).

Finally, the use of anesthetics in the present study might have an effect on the fluid movement. Indeed Xie L et al. showed that during anesthesia the drainage of extracellular fluid in mice is increased compared to the awake condition, but is similar to the results obtained during physiological sleep [17].

## Conclusion

**Fig. 8:**
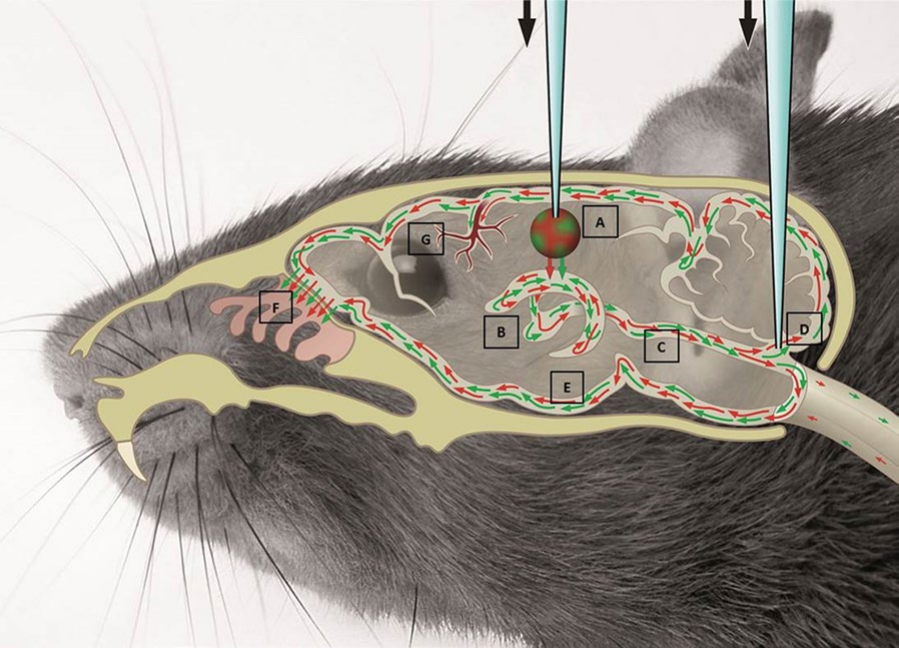
Overview of tracer distribution in a mouse head. Tracers injected in the striatum (*A*) distribute via ISF to the lateral ventricle (*B*) and subsequently to the ventricular system (*C*), where they mix with the CSF. From there, they spread along the ventricles and via the cisterna magna (*D*) they reach the SAS (*E*). Tracers leave the brain via the cribriform plate via the olfactory nerves (*F*). In the SAS tracers distribute along the leptomeningeal vessels, along the paravascular space (*G*)

The results of our study indicate that tracers injected into the striatum find their way to the ventricular system via ISF, dominated by bulk flow. The driving force for this flow could originate in parenchymal capillaries that act as a source of fluid production. In the ventricular system, ISF becomes mixed with CSF. Within the ventricles, the choroid plexus could play a dual role, not only producing CSF but also taking up low molecular weight substances. Since tracers injected into the cisterna magna did not enter the ventricular system, our data are consistent with the prevailing view that CSF flows along the ventricular system, reaches the subarachnoid space via the foramen of Magendie, and subsequently enters the subarachnoid space in the cisterna magna (Fig. 8) [33]. The relevance of this drainage pathway for removal of β-amyloid and other waste products remains to be explored.

## Additional files

10.1186/s12987-015-0019-5 Three dimensional reconstruction of the vasculature and distribution of tracers in a mouse head after injection in the striatum. Both TR-3 and F-500 entered the closest lateral ventricle. The tracers further dispersed to the contralateral ventricle, along the ventricular system, the subarachnoid space ventral of the olfactory bulb, and the nasal turbinates.
10.1186/s12987-015-0019-5 Three dimensional reconstruction of the vasculature and distribution of tracers in a mouse head after injection in the cisterna magna. Both TR-3 and F-500 were observed in the subarachnoid space, particularly on the ventral side of the brain around the circle of Willis. Both tracers left the brain through the cribriform plate and nasal turbinates.

## Abbreviations

ISF: interstitial fluid
CSF: cerebrospinal fluid
SAS: subarachnoid space
CM: cisterna magna
TR-3: dextran, Texas Red, 3 kD
F-500: dextran, FITC, 500 kD
BCSFB: blood-CSF barrier
